# Adjunct tele-yoga on clinical status at 14 days in hospitalized patients with mild and moderate COVID-19: A randomized control trial

**DOI:** 10.3389/fpubh.2023.1054207

**Published:** 2023-03-09

**Authors:** Vijaya Majumdar, N. K. Manjunath, Raghuram Nagarathna, Suryanarayan Panigrahi, Muralidhar Kanchi, Sarthak Sahoo, Hongasandra R. Nagendra, Adithi Giridharan, Mounika Reddy, Rakshitha Nayak

**Affiliations:** ^1^Swami Vivekananda Yoga Anusandhana Samsthana, Bengaluru, India; ^2^Narayana Health City, Bengaluru, India

**Keywords:** tele-yoga, COVID-19, hospitalized patients, clinical status, India

## Abstract

**Background:**

The initial insights from the studies on COVID-19 had been disappointing, indicating the necessity of an aggravated search for alternative strategies. In this regard, the adjunct potential of yoga has been proposed for enhancing the effectiveness of the standard of care with respect to COVID-19 management. We tested whether a telemodel of yoga intervention could aid in better clinical management for hospitalized patients with mild-to-moderate COVID-19 when complemented with the standard of care.

**Methods:**

This was a randomized controlled trial conducted at the Narayana Hrudyalaya, Bengaluru, India, on hospitalized patients with mild-to-moderate COVID-19 infection enrolled between 31 May and 22 July 2021. The patients (*n* = 225) were randomized in a 1:1 ratio [adjunct tele-yoga (*n* = 113) or standard of care]. The adjunct yoga group received intervention in tele-mode within 4-h post-randomization until 14 days along with the standard of care. The primary outcome was the clinical status on day 14 post-randomization, assessed with a seven-category ordinal scale. The secondary outcome set included scores on the COVID Outcomes Scale on day 7, follow-up for clinical status and all-cause mortality on day 28, post-randomization, duration of days at the hospital, 5th-day changes post-randomization for viral load expressed as cyclic threshold (Ct), and inflammatory markers and perceived stress scores on day 14.

**Results:**

As compared with the standard of care alone, the proportional odds of having a higher score on the 7-point ordinal scale on day 14 were ~1.8 for the adjunct tele-yoga group (OR = 1.83, 95% CI, 1.11–3.03). On day 5, there were significant reductions in CRP (*P* = 0.001) and LDH levels (*P* = 0.029) in the adjunct yoga group compared to the standard of care alone. CRP reduction was also observed as a potential mediator for the yoga-induced improvement of clinical outcomes. The Kaplan–Meier estimate of all-cause mortality on day 28 was the adjusted hazard ratio (HR) of 0.26 (95% CI, 0.05–1.30).

**Conclusion:**

The observed 1.8-fold improvement in the clinical status on day 14 of patients of COVID-19 with adjunct use of tele-yoga contests its use as a complementary treatment in hospital settings.

## Introduction

The rapid global spread of the coronavirus-related pneumonia outbreak, which was described first in December 2019, led to the evolution of one of the most extensive pandemics in human history so far (1–3). Although the mainstay of treatment for patients with COVID-19 pneumonia remains symptomatic and supportive care (4–6), the devastating impact of the pandemic led to a parallel unprecedented quest of identifying new and/or repurposed pharmacological treatments (5–10). Unfortunately, the initial indications from these studies were disappointing, which aggravated the search for strategies based on complementary and alternative medicine (5–11). Amid this uncertainty, several key clinicians and scientists identified and proposed the adjunct potential of yoga for enhancing the effectiveness of standard of care with respect to COVID-19 management in acute settings (12). The authors emphasized the relevance of certain practices of yoga and meditation in helping reduce the severity of COVID-19, including its collateral effects and sequelae (12), further underlining the immunomodulatory, anti-inflammatory, and stress modulatory potential of yoga (13–15). This notion was further strengthened by the findings of a preliminary report wherein tele-yoga intervention was reported to be safe, feasible, and useful in improving individual wellbeing and reducing stress (16). However, the long duration of direct exposure to patients during routinely delivered yoga interventions outweighs the benefit-to-risk ratio of physically delivered yoga interventions. Hence, we deemed the tele-mode of delivering the intervention as a viable and safer option for acute care in hospital settings. With the given background, this clinical trial was conducted to address the necessity of testing the effectiveness of tele-yoga as an adjunct to the standard of care in improving the clinical outcomes for adults hospitalized with COVID-19.

## Design and amendments

The protocol was approved by the Institutional Ethics Committee of Narayana Health City and conducted in compliance with the Declaration of Helsinki. The study protocol was approved for funding by the Department of Science and Technology, Government of India. All patients or legally authorized representatives provided written informed consent. Given the uncertainty in the recruitment and random allocation of the study subjects in chaotic hospital settings amid the pandemic, the trial was initially planned as a non-randomized clinical trial wherein an integrative yoga-based supportive care was planned to be administrated as an adjunct intervention for hospitalized patients with COVID-19. However, the protocol was amended on 14 May 2020, based on the emerging feasibility of conducting the randomization trial as emphasized by the clinicians due to the superior design of randomized vs. non-randomized trials. The study was registered at the clinical trial registry of India (CTRI/2020/09/027915, registered on 21/09/2020).

### Participants

Given a significant proportion of the requirement for timely hospitalization and management of patients with COVID-19, we recruited hospitalized patients with COVID-19 in this trial. Patients with mild and moderate COVID-19 were referred and managed at the Mazumdar Shaw Medical Center, Narayana Hrudyalaya, Bengaluru, India. SARS-CoV-2 cases, confirmed by polymerase chain reaction (PCR), were included as mild or moderate according to FDA guidance with the following eight symptoms: (17) fever, cough, sore throat, malaise, headache, muscle pain, gastrointestinal symptoms, and shortness of breath with exertion. Detailed eligibility criteria are listed below:

### Inclusion criteria

**The inclusion criteria are as follows:**

- Age 18–60 years old, both genders.
- Willing and able to provide written informed consent prior to performing study procedures.
- Oxygen saturation measured by pulse oximetry (SpO_2_) ≥ 90% on room air at sea level/with oxygen supplement non-invasively and not requiring intubation (18). The moderate disease definition was based on the SpO_2_ ≥ 90% and respiratory rate (RR) between 15 and 30/min.
- SARS-CoV-2 infection confirmed by PCR test ≤ 4 days before randomization.

### Exclusion criteria

**The exclusion criteria are as follows:**

- Breastfeeding and pregnant patients were excluded based on their declaration and pregnancy test results when required.
- Patients with SpO_2_ < 90% on room air, a ratio of arterial partial pressure of oxygen to the fraction of inspired oxygen (PaO_2_/FiO_2_) < 300 mm Hg, a respiratory rate of >30 breaths/min, or lung infiltrates of >50%.
- Patients diagnosed with critical COVID-19: respiratory failure, septic shock, and/or multiple organ dysfunction (MOD) or failure (MOF).
- Already enrolled in another COVID-19 trial or currently on any physiotherapy-based interventions.
- Unable to provide informed consent (e.g., moderate–severe dementia diagnosis).
- Those with more than 4 L per minute of supplemental oxygen (19).

### Outcomes

We used the seven-category ordinal scale that has been used in different COVID-19 therapeutic trials (7, 20). The primary outcome was the clinical status on day 14 post-randomization, assessed with a seven-category ordinal scale (the COVID Outcomes Scale) recommended by the World Health Organization (20). The scale consisted of seven mutually exclusive categories: 1, death; 2, hospitalized, receiving extracorporeal membrane oxygenation (ECMO) or invasive mechanical ventilation; 3, hospitalized, receiving noninvasive mechanical ventilation or nasal high-flow oxygen therapy; 4, hospitalized, receiving supplemental oxygen without positive pressure or high flow; 5, hospitalized, not receiving supplemental oxygen; 6, not hospitalized and unable to perform normal activities; and 7, not hospitalized and able to perform normal activities. To distinguish between categories 6 and 7, study personnel assessed the patient's performance of usual activities with questions consistent with validated health status measures (21).

All the patients provided written or electronic informed consent before randomization. The secondary outcome set included the following: scores on the COVID Outcomes Scale on day 7, follow-up for clinical status and all-cause mortality on the 28th-day post-randomization, duration of days at the hospital, 5th-day changes post-randomization for viral load expressed as cyclic threshold (Ct), and inflammatory markers and perceived stress scores on day 14. Other auxiliary markers were HbA1c, blood hemogram, and kidney function markers. All protocol amendments were authorized and approved by the institutional review board or independent ethics committee.

### Clinical and laboratory monitoring

#### Assessments

Data were collected daily, from randomization until day 28, in the patient proforma. For patients who were discharged before day 7, structured telephone calls were made to the patient or the family on days 7, 14, and 28 by an interviewer who was unaware of the assigned trial group to assess the vital status and return to routine activities. All samples were processed by PCR for genes N and E of SARS-CoV-2. Demographic, clinical, laboratory, and radiology data from patients' medical records were collected by the research team. The data were evaluated by a trained team of physicians. The date of disease onset was defined as the day when the symptom was noticed. Data on symptoms, vital signs, and laboratory values on biomarkers of disease progression, biomarkers [C-reactive protein (CRP), D-dimer, interleukin 6 (IL-6), ferritin, and lactate dehydrogenase (LDH)], and treatment measures during the hospital stay were collected. Patient assessments included physical examination, respiratory status (respiratory rate, type of oxygen supplementation, and blood oxygen saturation), adverse events, and concomitant medications. Blood-based investigations were done on days 1 and 5 post-randomization/hospitalization as per the routine analysis regime followed in the hospital settings. These investigations included measurement of blood cell counts, serum creatinine, glucose, total bilirubin, liver transaminases, and inflammatory biomarkers. Perceived stress was assessed using the Perceived Stress Scale 10 (PSS-10) (22). Site investigators assessed clinical status daily from days 1 to 14 or hospital discharge on a 7-point ordinal scale. The clinical status and mortality outcomes on the 28th day were assessed telephonically. In case of over a day change in the scores observed for the clinical status, worse scores of the hospitalized patients were documented. Final assessments on clinical status were done on day 28 personally for hospitalized patients or through telephonic interviews for already discharged patients.

### Intervention

We built a yoga protocol adjusted to isolated patients and staff, including delivery through tele-(videos) and in-person intervention. The recorded videos were used for the asynchronous delivery of tele-intervention. Instructional short videos were prepared in different languages constituting the intervention. While these videos were self-explanatory, yoga was delivered as supervised sessions with modules presented as recorded videos supervised by trained yoga therapists along with the distribution of practical training materials including both audio and video inputs. On day 1, the hands-on intervention was carried out in the COVID-19 wards through teams of certified yoga therapists in personal protective suites, within 4 h of randomization. The intervention was further continued in the hospital settings using tele-mode until discharge day using tele-(videos) along with facilitation through the physical presence of an instructor. The intervention was delivered daily two times for a duration of 10 min. For those who were discharged before 14 days post-randomization, tele-yoga sessions were continued from their home settings. Typical morning sessions were of 15-min duration and included flexibility exercises [hands in and out breathing (2 min), hands stretch breathing (2 min), and shoulder rotation (2 min) as part of their regular warm up]. These exercises were followed by quick relaxation and subsequent 8 min of pranayama (breathing exercises), consisting of abdominal breathing (3 min), alternate nostril breathing or Nadishuddhi pranayama (3 min), and Bhramari pranayama (2 min). These practices have been reported to have effects on the strengthening of the respiratory muscles, and respiratory function, including the development of awareness of expansion and contraction of the airways and continuous and rhythmic breathing, which has been reported to aid in thorough oxygenation of the lungs and reduce inflammation. The practice sessions ended with guided relaxation of 2 min with a resolve. Evening sessions were 10 min and focused on the aforementioned breathing exercises and concluded with guided meditation.

Clinical guidelines were followed up for treating patients via tele-yoga and hands-on techniques in cooperation with the medical heads of departments. The instructor or therapist monitored and ensured that the practices were done as per the module protocol and corrected the patients along with the doubt clarifications.

### Standard of care

The standard of care was based on the recommendations of the Indian Council of Medical Research, which was updated as per the evolving evidence generated in drug trials and international consensus guidelines (23, 24). Overall, it included antibiotic agents, antiviral agents, corticosteroids, vasopressor support, and anticoagulants at the discretion of the clinicians.

### Randomization

Randomization was done in permuted blocks of four in sequences created by the unblinded research staff in Microsoft Excel version 19.0 who provided masked allotment to the yoga trainers. Owing to the nature of the intervention, blinding was not possible, but outcome measures were blinded for the randomization groups. Eligible patients were randomly assigned in a 1:1 ratio to receive either standard of care or adjunct yoga. Allocation assignment was concealed from investigators and patients.

### Statistical analysis

#### Sample size calculation

The sample size of 230 patients with a 1:1 randomization of adjunct tele-yoga to the standard of care provides ~80% power to detect a 15% difference between treatment groups in time cumulative hospital discharge (i.e., with or without limiting abilities) rates of 80% in the adjunct tele-yoga group and 75% in the standard of care group, on day 14, using a two-sided 5% alpha. Analysis was performed with SPSS version 23 [IBM Corp., (N.Y., USA)].

The trial was analyzed by comparing patients randomized to adjunct tele-yoga vs. those randomized to standard of care, with the placebo group serving as the referent. The primary outcome was analyzed with a multivariable proportional odds model adjusted with age, sex, and comorbidities. Further adjustments with baseline (pre-randomization) COVID Outcomes Scale category and duration of acute respiratory symptoms are reported as *post-hoc* analysis. The results are presented with corresponding 95% confidence intervals. As mentioned earlier, for patients who were discharged prior to 7 days after randomization, primary outcome ascertainment was completed by telephone calls. Patients who could not be reached by telephone for the primary outcome assessment on days 7, 14, or 28 had the COVID Outcomes Scale score carried forward from the last outcome follow-up call if such a call was successfully completed or had a category 6 score (not hospitalized and unable to perform normal activities) imputed if no prior follow-up calls were successfully completed (8). For patients who remained hospitalized 14 days after randomization, primary outcome ascertainment was completed by a review of the medical records. Given the deviation from normality for the study variables, analysis of covariance was done using the rank transformation to study the influence of adjunct tele-yoga intervention on biomarker levels. A *p*-value of <0.05 was considered to indicate significant differences.

The heterogeneity of treatment effect by prespecified baseline characteristics was evaluated by adding an interaction term between randomized group assignment and the baseline characteristic of interest in the primary model. Baseline characteristics evaluated in the heterogeneity of treatment effect analyses included baseline COVID Outcomes Scale category, and duration of symptoms prior to randomization, age, sex, and race/ethnicity.

All-cause mortality was estimated using the Kaplan–Meier product limit method. The adjunct tele-yoga group was compared with the standard of care group using the log-rank test, and the mean estimates and 95% CIs were provided.

We also used the paramed command in SPSS to perform mediation analysis by fitting a linear regression model to the outcomes with yoga treatment, and the mediators included were the covariates. We applied the causal mediation method, to investigate whether biomarkers could be a causal pathway between intervention and the outcome. Regression models were fitted to the mediators with treatments included as covariates.

### *Post-hoc* analyses

We also conducted sensitivity analyses of the primary endpoint adjusting for the day 1 clinical score and duration of symptoms. In addition, given the imbalance in the proportion of breathlessness at the baseline, we further analyzed the primary outcome with breathlessness prevalence as a covariate in the ordinal regression model. We also performed a *post-hoc* analysis that was stratified by CRP and LDH levels. We also calculated and compared the proportions of patients with a one-point or greater improvement, no change, or worsening of clinical status on days 7, 14, and 28.

## Results

**Figure 1:**
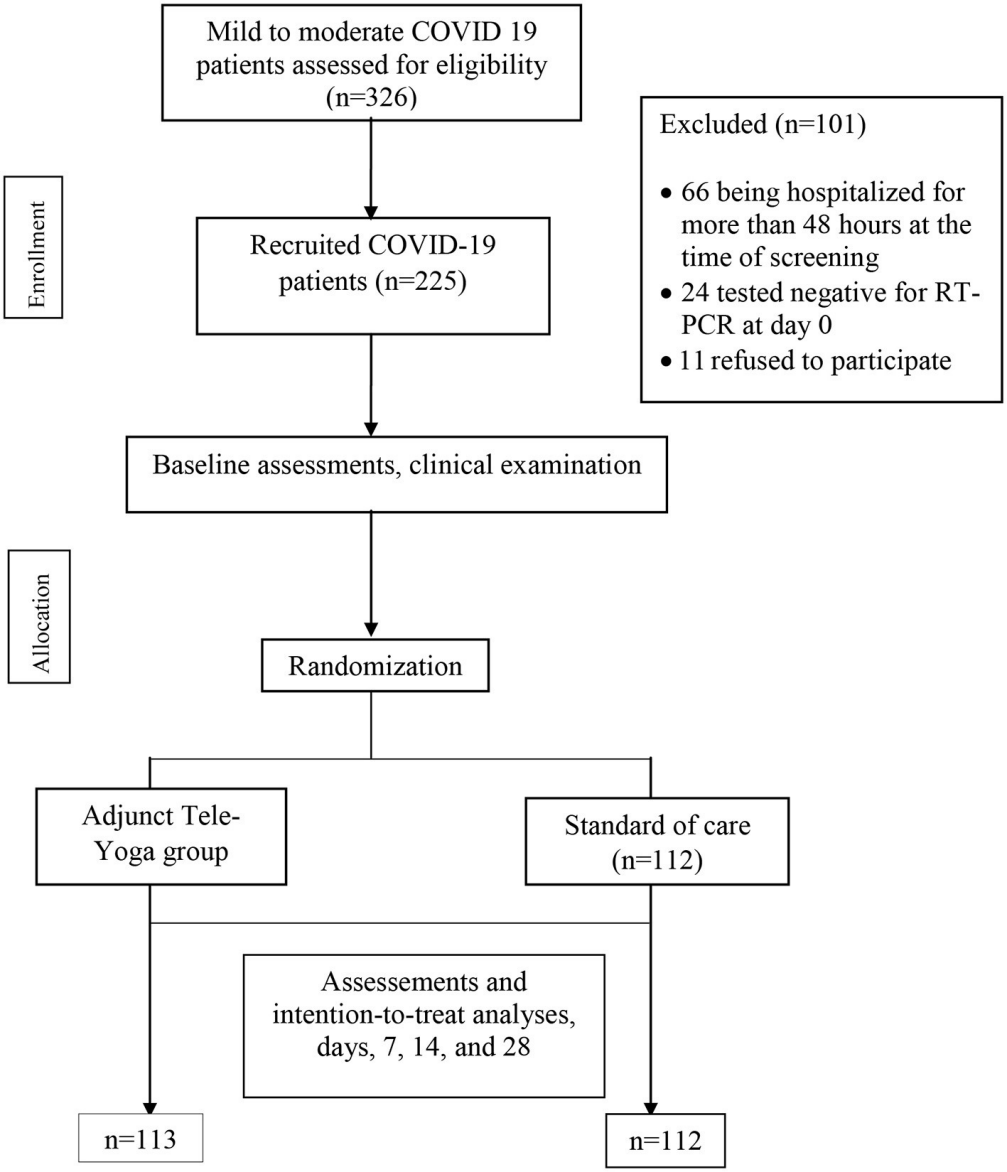
Trial profile.

During the 60 days enrollment period **(**31 May and 22 July 2021), 326 patients were screened, 66 (20.24%) patients were excluded for being hospitalized for more than 48 h at the time of screening, and 24 (7.36%) had tested negative for RT-PCR on day 0 (baseline) for COVID-19. Furthermore, 11 eligible patients refused to participate (2.76%) (see Figure 1). Hence, out of 326 eligible patients, 225 could be randomized, 113 were randomized to the adjunct tele-yoga, and 112 were randomized to the standard of care group. The last outcome assessment was on 31 July 2021. Demographics and baseline disease characteristics of participants in both groups are presented in Table 1. Overall, the median age of the participants was 43 years (IQR, 35–53 years), 54.67% were male subjects, 37.78% had diabetes, 20.89% had hypertension, and 6.67% had coronary artery disease. There was an equal distribution of age, gender, and days before the onset of symptoms, comorbidities, and inflammatory markers between the study arms (Table 1; Supplementary Table S2). Overall, at baseline, 70.22% of patients presented with perceived or objective fever, 72.44% presented with cough, 12.44% presented with a sore throat, 25.33% presented with headache, and 50.72% presented with breathlessness. Other than the prevalence of breathlessness (*P* = 0.006), there were no remarkable differences between groups with respect to the distribution of covariates. The median duration of symptoms prior to randomization was 3 days (IQR, 2–4 days) in both groups. There were no differences in either vital signs or full blood count between the groups (Supplementary Table S2). Of the 113 patients in the adjunct yoga group, 29 (76%) were discharged before 7 days post-randomization and thus continued with tele-yoga sessions until the 14th day.

**Table 1:** Baseline patient characteristics.

**Variable**	**Overall (*n* = 225)**	**Tele—yoga (*n* = 113)**	**Control (*n* = 112)**	***P*-value**
Age, median IQR	43 (35–53)	42 (35–53.5)	43 (36–52)	0.657
**Sex**				
Female	102 (45.33)	51 (45.13)	51 (45.54)	1.00
Male	123 (54.67)	62 (54.87)	61 (54.46)	
**Coexisting conditions**				
Hypertension, *n* (%)	47 (20.89)	21 (18.58)	26 (23.21)	0.416
Diabetes, *n* (%)	85 (37.78)	42 (37.17)	43 (38.39)	0.891
Coronary artery disease, *n* (%)	15 (6.67)	5 (4.43)	10 (8.93)	0.193
Hypothyroidism, *n* (%)	25 (11.11)	14 (12.39)	11 (9.82)	0.672
COPD, *n* (%)	3 (1.33)	3 (2.65)	0 (0)	0.222
Asthma, *n* (%)	2 (0.89)	0	2 (1.80)	0.244
**Symptoms**				
Fever/chills, *n* (%)	158 (70.22)	73 (64.61)	85 (75.89)	0.080
Cough, *n* (%)	163 (72.44)	82 (72.57)	81 (72.32)	1.000
Sore throat, *n* (%)	28 (12.44)	16 (14.16)	12 (10.71)	0.545
Nausea/vomiting, *n* (%)	13 (5.78)	7 (6.19)	6 (5.36)	1.000
General weakness, *n* (%)	92 (40.89)	48 (42.48)	44 (39.28)	0.685
Breathlessness, *n* (%)	105 (50.72)	44 (41.90)	61 (59.80)	0.006^**^
Headache, *n* (%)	57 (25.33)	34 (30.09)	23 (20.54)	0.125
Diarrhea , *n* (%)	11 (5.31)	4 (3.81)	7 (6.86)	NS
**Previous medication use—no. (%)**				
Glucocorticoid	7 (3.03)	5 (4.35)	2 (1.67)	NS
ACE inhibitor	12 (5.19)	7 (6.19)	5 (4.46)	NS
Angiotensin II–receptor antagonist	8 (3.46)	3 (2.61)	5 (4.35)	NS
**Baseline ordinal COVID outcome score—no. (%)**				
3. Hospitalized, not receiving supplemental oxygen	92 (40.89)	54 (47.79)	38 (33.93)	0.60
4. Hospitalized, receiving supplemental oxygen without positive pressure or high flow; requiring low-flow supplemental oxygen	125 (55.56)	57 (50.44)	68 (60.71)	
5. Hospitalized, receiving non-invasive ventilation or high-flow nasal cannula	8 (3.56)	2 (1.77)	6 (5.36)	
Ct value	28.00 (22.5–32.00)	27.00 (22.50–30.00)	28.0 (22.50–33.00)	0.125
**Inflammatory markers**				
C-reactive protein, mg/l	24.82 (8.09–63.67)	28.16 (8.43–65.46)	26.71 (8.47–67.40)	0.854
Ferritin, ng/ml	196 (81.85–421)	179 (82.30–404.50)	203 (77.40–441)	0.616
D-dimer, ng/ml	167 (94.00–242.00)	170 (94–245)	179 (95–250)	0.953
LDH, U/l	302 (241–392)	296 (226.50–355)	319 (248–436.94)	0.057
IL-6, mg/dl	37.65 (11.27–80.02)	31.89 (11.93–79.99)	39.76 (10.21–76.15)	0.808
**Haemogram**				
Hemoglobin (g/dl)	13.50 (12.20–14.60)	13.6 (12.10–14.70)	13.2 (12.20–14.45)	0.406
ALC (×10^9^/L)	1.27 (0.87–1.92)	1.21 (0.84–1.86)	1.34 (0.88–1.95)	0.472
AMC (×10^9^/L)	0.46 (0.29–0.74)	0.45 (0.28–0.72)	0.50 (0.32–0.77)	0.343
ANC (×10^9^/L)	4.23 (2.85–6.71)	4.17 (2.91–6.64)	4.39 (2.73–6.83)	0.606
PSS	19 (15–24)	20 (16–25)	19 (13.25–23)	0.023^*^

For continuous variables, median and interquartile range (IQR) have been presented due to the non-normality of the data. Correspondingly, Mann–Whitney tests were used to assess whether differences between the study groups were statistically significant. For categorical variables, chi-square/Fisher's exact tests were used to check whether there was any association between the groups. Ct, cyclic threshold value; ALC, absolute lymphocyte count; AMC, absolute monocyte count; ANC, absolute neutrophil count; PSS, Perceived Stress Scale.

* *P* < 0.05;

** *P* < 0.001; NS, Not significant.

### Primary outcome

**Figure 2:**
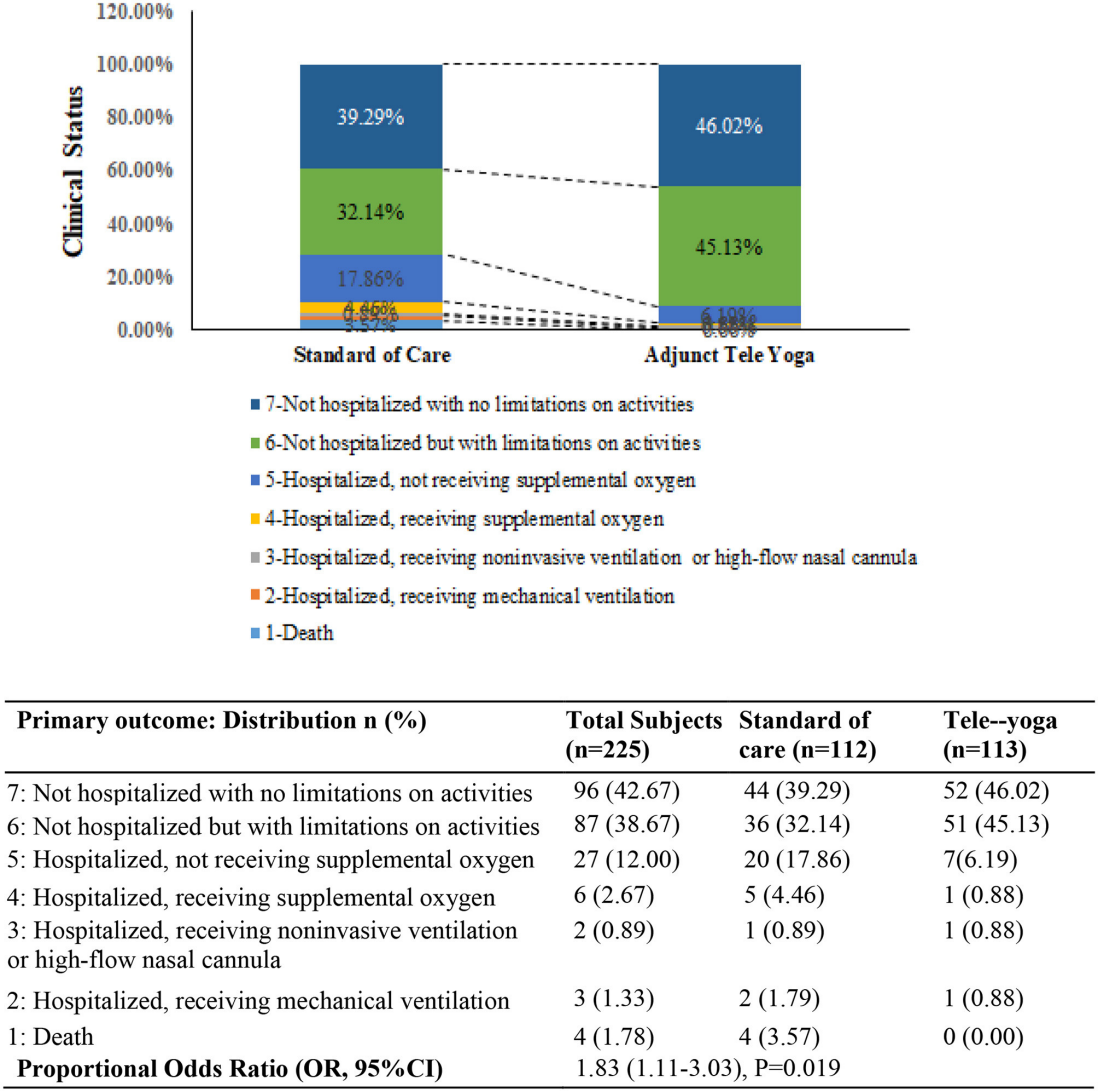
Clinical status on the coronavirus disease (COVID) outcomes scale 14 days. The primary outcome was assessed in all patients who were still in the hospital on day 14 exactly and in outpatients (by means of telephonic interview) as close to day 14 as possible. OR—odds ratio was derived from the multivariable proportional odds model adjusted for baseline age, sex, and comorbidities (diabetes, hypertension, and hypothyroid). *P*-value <0.05 was considered significant.

For the analysis of outcomes, 113 and 112 patients were included for the adjunct tele-yoga and the standard of care groups, respectively; the analysis was by the originally assigned groups. The primary outcome (status on the 7-point ordinal scale on day 14) was assessed in all patients who were still hospitalized on day 14 or who were telephonically interviewed after being discharged from the hospital. The distribution of patients' scores on the seven-level ordinal scale at 14 days is shown in Figure 2. Patients randomized to the adjunct tele-yoga group had significantly higher odds of a better clinical status distribution on the 7-point ordinal scale compared with those randomized to standard care (odds ratio, 1.83, 95% CI = 1.11–3.03) (Figure 2). The model was adjusted for age, sex, and comorbidities. Sensitivity analyses of the primary endpoint adjusting for day 1 clinical status score and symptom duration using the intention-to-treat population produced no significant difference (Supplementary Table S3). Given the imbalance in the baseline distribution, we additionally adjusted the model for the prevalence of breathlessness, which did not lead to any significant reduction in effect size (Supplementary Table S3) (odds ratio, 1.84, 95% CI = 1.09–3.12). The results for the primary outcome were not different across the prespecified subgroups (Supplementary Table S4).

### Secondary outcomes

**Figure 3:**
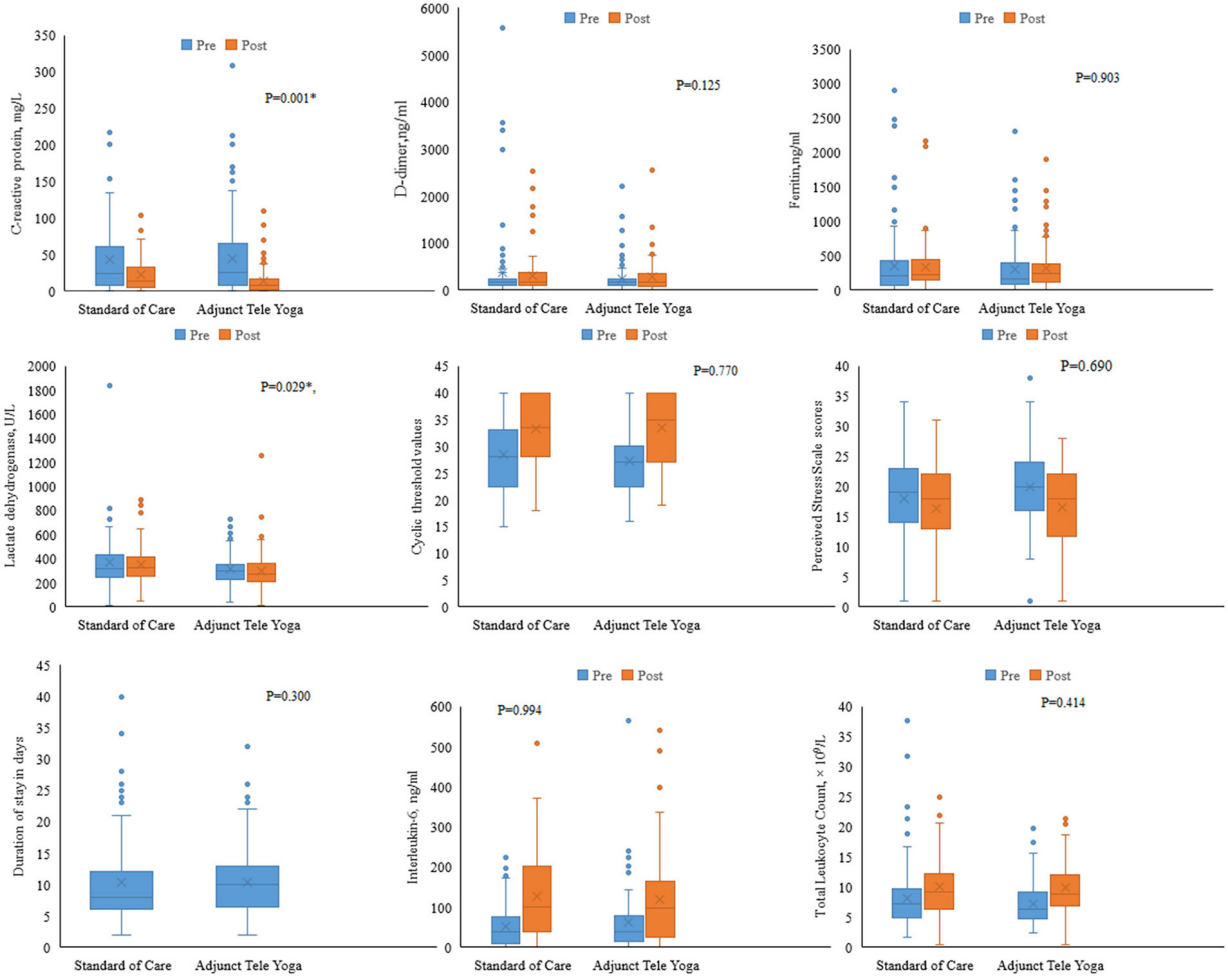
Biomarker levels on day 5 post-randomization. Changes in the biomarkers on day 5 were analyzed with respect to the baseline values. Analysis of covariance was done using the rank transformation to study the influence of adjunct tele-yoga intervention on biomarker levels. *Indicates *P*-value <0.05.

There were significant differences between the adjunct tele-yoga and standard care groups in terms of improvement in clinical status on the 7th day (partially adjusted for age odds ratio, 3.61; 95% CI, 2.11–6.05; *P* < 0.001), but the follow-up outcome on 28th day was not significant (adjusted odds ratio, 1.70% CI = 1.03–3.44) (Supplementary Table S5). On day 5, there were significant reductions in CRP (*p* = 0.001) and LDH levels (*P* = 0.029) in the adjunct yoga group compared to the standard of care alone (Figure 3; Supplementary Table S6). There were no significant differences between the treatment groups in the duration of hospitalization, viral load (cyclic threshold values), and other markers of inflammation such as ferritin and D-dimer (Figure 3). The Kaplan–Meier estimates of all-cause mortality on day 28 were 1.80 vs. 5.40% for the standard of care [log-rank *P*-value = 0.144; adjusted hazard ratio (HR), 0.26; 95% CI, 0.05–1.30] (Supplementary Figure S1).

### Exploratory outcomes

Since we could establish significant reductions in CRP and LDH on day 5 from post-randomization in the adjunct yoga group compared to the standard of care group alone, we further tested for their mediating effects on the intervention (Table 2). The analyses indicated CRP as a potential mechanistic mediator of adjunct yoga on the improved clinical status on the 14th day post-intervention. There were also differences between proportions of subjects with at least 1 unit change in outcomes on day 7 from baseline between adjunct tele-yoga as compared to the standard of care groups. However, the distributions were not different for days 14 and 28 (Supplementary Figure S2).

**Table 2:** Indirect, direct, and total effects of the mediation models on COVID-19 outcomes at 14-day post-randomization.

	**Effect size**	**Proportion mediated**
Direct effect of the adjunct tele-yoga vs. standard of care adjunct yoga	0.41 (0.03–0.78)	–
Total effect of the model	0.54 (0.17–0.91)	–
**Indirect (mediating) effects**		
LDH	−0.01 (−0.10 to 0.04)	Not significant
CRP	0.06 (0.05–0.16)^*^	11.11%

LDH, lactate dehydrogenase; CRP, C-reactive protein.

* *P* < 0.05.

Direct effect measures the direct influence of the intervention on the primary outcome that is not mediated by other variables in the model. An indirect or mediated effect expresses the portion of the intervention effect that is mediated through a specific mediator. The total effect is the sum of the direct and indirect effects of the BFY and mediators on the study outcomes.

### Adverse effects

None of the eight deaths through day 28 [five (1%) in the standard of care, and three (2%) in the adjunct tele-yoga group] occurred in the patients with COVID-19 could be attributed to the tele-yoga intervention (Supplementary Table S7). In the tele-yoga group, the extension of hospitalization was 10.62%, whereas in the standard of care alone it was (21) 18.75%. Single cases of sinus tachycardia and pulmonary embolism were observed in the yoga group as compared to no cases in the standard of care.

## Discussion

This study is a pioneer clinical trial that investigated the short-term acute interventional benefits of adjunct tele-yoga practice for the clinical management of hospitalized patients with COVID-19. We could establish a ~1.9-fold improvement in the clinical status on the 14th day, in hospitalized patients with mild and moderate COVID-19 (odds ratio = 1.83, 95% CI = 1.11–3.03) as compared to those with the only standard of care. The odds of improvement with yoga intervention were higher on the 7th day (odds ratio = 3.61, 95% CI = 2.13–6.10). However, the effectiveness of the intervention was not found to be sustained at the 28th-day follow-up (odds ratio 1.70, 95% CI = 0.97–2.99, *P* = 0.07). Since patients had several coexisting diseases and were subjected to a diverse medication regimen, the complementary effects of tele-yoga could have been influenced by the heterogeneity of the sample and its treatment. However, when analyzed in the *post-hoc* subgroup analysis, adjunct yoga was found to be effective across all the strata of covariates. Concerning the influence of the intervention on mortality-related outcomes, no benefit could be observed for the adjunct yoga intervention with respect to mortality (hazards ratio = 0.26; 95% CI, 0.05–1.30). However, we could establish support for the primary endpoints with the observed secondary improvement in crucial biomarkers in the tele-yoga group compared to the standard of care on 5th-day post-randomization, CRP (*P* = 0.001) and LDH (*P* = 0.029). Both CRP and LDH have been reported as prognostic markers of deterioration in patients with COVID-19 including mild/non-severe cases as well (25, 26). We could also establish a mediation effect of CRP modulation underlying the effectiveness of tele-yoga intervention (~11% proportion mediation on the observed improved outcome of clinical status on day 14). This inflammation-reducing effect of yoga well-aligns with the physiological modulation of vagal tone, one of the widely reported effects of yoga and meditation (12, 13). The anti-inflammatory potential of yoga could serve as a step forward in the fight against other serious forms of infectious diseases with a dominant inflammatory component, as proposed for malaria, HIV/ AIDS, and SARS, among others. However, there was no significant modulating influence of the adjunct yoga intervention observed on other prognostic markers of COVID-19, in particular D-dimer and ferritin levels, which could be explained by their not so deregulated status at the baseline (D-dimer, median = 167 ng/ml (IQR, 94.00–242.00), and Ferritin levels, median = 196 ng/ml (IQR, 81.85–421).

We could not observe a significant effect of adjunct tele-yoga on the Perceived Stress Scale in patients with COVID-19 (*P* = 0.69). We speculate that the failure to obtain the desired effect on stress and several other variables could be due to the primarily virtual mode of the delivery of the intervention and the short duration of the intervention. However, the beneficial clinical outcomes observed in the study hold special significance in the present era with reemerging and recurring viral infections (27, 28). Overall, the findings of this study support the exploratory notions of several researchers and clinicians that certain meditation, yoga asana (postures), and pranayama (breathing) practices may be effective adjunctive means of treating SARS-CoV-2 infection (12). The findings also pave the foundation for the clinical implementation of tele-yoga-based adjunct interventions in hospital settings for the management of infectious diseases. A previous study on yoga had also reported it to be effective as an adjunct to anti-tuberculosis treatment (ATT) in patients with pulmonary tuberculosis by reducing the symptom scores, sputum conversion on microscopy, and improvement in the lung capacity and radiographic pictures (29).

This clinical exploration is one of the earliest to be reported among several other concomitant attempts to establish the efficacy of additional systems of medicine, against the combat of COVID-19, as evidenced by 67 such registered trials in the Clinical Trial Registry of India (CTRI) (30). Hence, given the lack of available findings from clinical trials on COVID-19 and yoga-based interventions, the findings of this trial could not be presented with comparisons.

The study has several strengths. One of the strengths of the study is the inclusion of WHO criteria for assessing the benefit on clinical status for patients hospitalized with mild and moderate COVID-19. This is the first report wherein yoga-based intervention was provided in a tele-mode to patients with COVID-19. This was done to prevent healthcare employees from being infected. Importantly, the trial included inflammatory markers as study outcomes, wherein an anti-inflammatory mediating influence of yoga intervention could be established to improve the outcomes of hospitalized patients with mild-to-moderate COVID-19. A key feature of the trial was the early implementation of treatment within 7 days of symptom onset (median duration of 3 days) which has been considered important for the treatment protocol, in particular antivirals such as remdesivir.

The trial was limited to hospitalized patients with COVID-19 which restricts the generalizability of the findings to other populations involving home-based care. The intervention duration was limited to 14 days, and assessments were limited to 28 days follow-up; however, the continued intervention could have led to sustained positive effects with respect to late complications of COVID-19. Reporting long-term outcomes of trial participants should have been considered. Given the nature of the intervention, the study used an open-label design, which could have led to biases in patient care and reporting of data. Due to logistic challenges, the laboratory-based parameters could not be collected on the prespecified 14th day time point. There was also an imbalance in the baseline distribution of the covariate, breathlessness, indicating differences in the severity status of the subjects between groups. However, we confirmed the robustness of the primary outcome with a *post-hoc* analysis adjusting for the baseline distribution of the covariate in the ordinal regression model.

Overall, we could observe clinically relevant effects among hospitalized patients with mild-to-moderate COVID-19, contesting the use of tele-yoga as a complementary treatment for patients with this disease. However, the positive signal found in this small-scale trial warrants the conduction of larger trials using tele-yoga for the treatment of COVID-19.

## Data availability statement

Datasets are available on request to the corresponding author.

## Ethics statement

The study protocol was approved by the Institutional Ethics Committees of the Narayana Health City and Swami Vivekananda Yoga Anusandhana Samsthana, Bengaluru, India. The patients provided their written informed consent to participate in this study.

## Author contributions

VM, RNag, and NM take responsibility for the integrity of the data and the accuracy of the data analysis and drafting of the manuscript. RNag, NM, and VM contributed to concept and design. SP, SS, AG, MR, RNay, and VM contributed in acquisition, analysis, or interpretation of data. NM, RNag, MK, and HN did critical revision of the manuscript for important intellectual content. VM contributed in statistical analysis. VM, NM, and RNag obtained funding and contributed in administrative, technical, or material support. All authors contributed to the article and approved the submitted version.

## References

[1] Dong E Du H Gardner L. An interactive web-based dashboard to track COVID-19 in real time [published correction appears in Lancet Infect Dis. 2020 Sep;20:e215]. Lancet Infect Dis. (2020) 20:533–4. 10.1016/S1473-3099(20)30120-1 32087114 PMC7159018

[2] Phelan AL Katz R Gostin LO. The novel coronavirus originating in Wuhan, China: challenges for global health governance. JAMA. (2020) 323:709–10. 10.1001/jama.2020.1097 31999307

[3] World Health Organization. COVID-19 Public Health Emergency of International Concern (PHEIC) Global Research and Innovation Forum. (2020). Available online at: https://www.who.int/publications/m/item/covid-19-public-health-emergency-of-international-concern-(pheic)-global-research-and-innovation-forum (accessed November 10, 2022).

[4] Sanders JM Monogue ML Jodlowski TZ Cutrell JB. Pharmacologic treatments for coronavirus disease 2019 (COVID-19): a review. JAMA. (2020) 323:1824–36. 10.1001/jama.2020.6019 32282022

[5] Fan E Beitler JR Brochard L Calfee CS Ferguson ND Slutsky AS. COVID-19-associated acute respiratory distress syndrome: is a different approach to management warranted? Lancet Respir Med. (2020) 8:816–21. 10.1016/S2213-2600(20)30304-0 32645311 PMC7338016

[6] Cusinato J Cau Y Calvani AM Mori M. Repurposing drugs for the management of COVID-19. Expert Opin Ther Pat. (2021) 31:295–307. 10.1080/13543776.2021.1861248 33283567

[7] Spinner CD Gottlieb RL Criner GJ López JRA Cattelan AM Viladomiu AS. Effect of remdesivir vs standard care on clinical status at 11 days in patients with moderate COVID-19: a randomized clinical trial. JAMA. (2020) 324:1048–57. 10.1001/jama.2020.16349 32821939 PMC7442954

[8] Self WH Semler MW Leither LM Casey JD Angus DC Brower RG. Effect of hydroxychloroquine on clinical status at 14 days in hospitalized patients with COVID-19: a randomized clinical trial. JAMA. (2020) 324:2165–76. 10.1001/jama.2020.22240 33165621 PMC7653542

[9] López-Medina E López P Hurtado IC Dávalos DM Ramirez O Martínez E. Effect of ivermectin on time to resolution of symptoms among adults with mild COVID-19: a randomized clinical Trial. JAMA. (2021) 325:1426–35. 10.1001/jama.2021.3071 33662102 PMC7934083

[10] Siemieniuk R. A. Bartoszko J. J. Ge L. Zeraatkar D. Izcovich A. Kum E.. Drug treatments for covid-19: living systematic review and network meta-analysis [published correction appears in BMJ. (2021) 373:n967]. BMJ. (2020) 370:m2980. 10.1136/bmj.m2980 32732190 PMC7390912

[11] Murthy S Gomersall CD Fowler RA. Care for critically ill patients with COVID-19. JAMA. (2020) 323:1499–500. 10.1001/jama.2020.3633 32159735

[12] Bushell W Castle R Williams MA Brouwer KC Tanzi RE Chopra D. Meditation and yoga practices as potential adjunctive treatment of SARS-CoV-2 infection and COVID-19: a brief overview of key subjects. J Altern Complement Med. (2020) 26:547–56. 10.1089/acm.2020.0177 32579021

[13] Bower JE Irwin MR. Mind-body therapies and control of inflammatory biology: a descriptive review. Brain Behav Immun. (2016) 51:1–11. 10.1016/j.bbi.2015.06.012 26116436 PMC4679419

[14] Barrett B Hayney MS Muller D Rakel D Brown R Zgierska AE. Meditation or exercise for preventing acute respiratory infection (MEPARI-2): a randomized controlled trial. PLoS ONE. (2018) 13:e0197778. 10.1371/journal.pone.0197778 29933369 PMC6014660

[15] Obasi CN Brown R Ewers T Barlow S Gassman M Zgierska A. Advantage of meditation over exercise in reducing cold and flu illness is related to improved function and quality of life. Influenza Other Respir Viruses. (2013) 7:938–44. 10.1111/irv.12053 23170828 PMC3582749

[16] Jasti N Bhargav H George S Varambally S Gangadhar BN. Tele-yoga for stress management: need of the hour during the COVID-19 pandemic and beyond? Asian J Psychiatr. (2020) 54:102334. 10.1016/j.ajp.2020.102334 32777755 PMC7396129

[17] Food Drug Administration. COVID-19: Developing Drugs and Biological Products for Treatment or Prevention. (2020). Available online at: https://www.fda.gov/regulatory-information/search-fda-guidance-documents/covid-19-developing-drugs-and-biological-products-treatment-or-prevention (accessed August 23, 2021).

[18] Ministry of Health and Family Welfare. Government of India. Guidance Document on Appropriate Management of Suspect/Confirmed Cases of COVID-19. Available online at: https://www.mohfw.gov.in/pdf/FinalGuidanceonMangaementofCovidcasesversion2.pdf (accessed December 1, 2020).

[19] Cavalcanti AB Zampieri FG Rosa RG Azevedo LC Veiga VC Avezum A. Hydroxychloroquine with or without azithromycin in mild-to-moderate COVID-19. N Engl J Med. (2020) 383:e119. 10.1056/NEJMx200021 32706953 PMC7397242

[20] World Health Organization. WHO R&D Blueprint: Novel Coronavirus: COVID-19 Therapeutic Trial Synopsis. Available online at: https://www.who.int/blueprint/priority-diseases/key-action/COVID-19_Treatment_Trial_Design_Master_Protocol_synopsis_Final_18022020.pdf (accessed June 28, 2020).

[21] EuroQol Group. EuroQol: a new facility for the measurement of health-related quality of life. Health Policy. (1990) 16:199–208. 10.1016/0168-8510(90)90421-9 10109801

[22] Cohen S Kamarck T Mermelstein R. A global measure of perceived stress. J Health Soc Behav. (1983) 24:385–96. 10.2307/2136404 6668417

[23] Government of India. Ministry of Health & Family Welfare. Clinical Management Protocol: COVID-19. Available online at: https://www.mohfw.gov.in/pdf/ClinicalManagementProtocolforCOVID19.pdf (accessed December 1, 2020).

[24] Malhotra V Basu S Sharma N Kumar S Garg S Dushyant K. Outcomes among 10,314 hospitalized COVID-19 patients at a tertiary care government hospital in Delhi, India. J Med Virol. (2021) 93:4553–8. 10.1002/jmv.26956 33755238 PMC8251427

[25] Wang G Wu C Zhang Q Wu F Yu B Lv J. C-reactive protein level may predict the risk of COVID-19 aggravation. Open Forum Infect Dis. (2020) 7:ofaa153. 10.1093/ofid/ofaa153 32455147 PMC7197542

[26] Shi J Li Y Zhou X Zhang Q Ye X Wu Z. Lactate dehydrogenase and susceptibility to deterioration of mild COVID-19 patients: a multicenter nested case-control study. BMC Med. (2020) 18:168. 10.1186/s12916-020-01633-7 32493370 PMC7268591

[27] Abrahão JS de Arruda LB. Special issue “emerging viruses: surveillance, prevention, evolution, and control”. Viruses. (2020) 12:306. 10.3390/v12030306 32168932 PMC7150905

[28] Karim SSA Karim QA. Omicron SARS-CoV-2 variant: a new chapter in the COVID-19 pandemic. Lancet. (2021) 398:2126–8. 10.1016/S0140-6736(21)02758-6 34871545 PMC8640673

[29] Visweswaraiah NK Telles S. Randomized trial of yoga as a complementary therapy for pulmonary tuberculosis. Respirology. (2004) 9:96–101. 10.1111/j.1440-1843.2003.00528.x 14982609

[30] Umesh C Ramakrishna KK Jasti N Bhargav H Varambally S. Role of ayurveda and yoga-based lifestyle in the COVID-19 pandemic - a narrative review. J Ayurveda Integr Med. (2022) 13:100493. 10.1016/j.jaim.2021.07.009 34305355 PMC8286865

