# Supplementary material for: Adjunct tele-yoga on clinical status at 14 days in hospitalized patients with mild and moderate COVID-19: A randomized control trial
Source: Front Public Health. 2023 Mar 9;11:1054207. doi: 10.3389/fpubh.2023.1054207 (PMC10034105; doi:10.3389/fpubh.2023.1054207)
Supplement: Supplementary file 1 [file Data_Sheet_1.docx]

**TABLE OF CONTENTS**

1. VALIDATION OF YOGA MODULE,
2. ADDITIONAL RESULTS

TABLE S2. ADDITIONAL CHARACTERISTICS OF THE POPULATION AT BASELINE

TABLE S3. POST-HOC SENSITIVITY ANALYSES FOR THE PRIMARY ENDPOINT

TABLE S4. SUBGROUP ANALYSES OF THE PRIMARY OUTCOME

FIGURE S1. SURVIVAL THROUGH 28 DAYS FOLLOWING RANDOMIZATION

TABLE S5: CLINICAL STATUS OUTCOMES (INTENTION-TO-TREAT POPULATION)

TABLE S6. BIOMARKER LEVELS AT DAY 5 POST RANDOMIZATION

FIGURE S2. CHANGE IN CLINICAL STATUS OVER TIME

TABLE S7. ADVERSE EVENTS

TABLE S8. CONCOMITANAT MEDICATIONS DURING HOSPITAL STAY

1. MEDIATION MODEL

**APPENDIX I. VALIDATION OF INTEGRATIVE YOGA MODULE FOR COVID-19**

The first objective of the trial was to develop an effective intervention module, hence, a module was drafted which was derived from the research evidence available on the antiviral, anti-inflammatory, distressing and respiratory aspects of yoga. The chosen practices have been reported develop the awareness of expansion and contraction of the airways, make breathing uniform, continuous and rhythmic, and aid in uniform and thorough oxygenation of the lungs, opening of blocked air passages, with a balancing effect on bronchial reactivity, and improve respiratory function. Hence, there was a focus on the integration of practices to confer a holistic treatment aiming for: relaxation, breath regulation and strengthening of respiratory muscles through breathing practices, and coping with emotional distress. We drafted the intervention, with texts and pictorials. These exercises were drafted to be followed by quick relaxation and subsequent 10 minutes of pranayama (breathing exercises), that in turn would comprise right nostril breathing/and alternate nostril breathing and Bhramari Pranayama (humming). Pranayama is a term that is associated with Astanga Yoga and deals with formal and traditional practice of control of breath and is intended for regulation of prana-the life-force . Pranayama is controlling the breathing process in a therapeutic way, leading to improvement of the pulmonary reserve function, and efficient neurological control at a basic and obvious level. The Bhramari Pranayama is presented for possible lifestyle interventions to reduce the risk of infection, increase lung function, enhance autonomic function, and improve sleep quality in healthy individuals. The practice sessions were further aimed to end with guided relaxation with a resolve. Towards the validation of the drafted module, we included 25 Yoga experts (25 responders). These highly qualified and experienced Yoga practitioners and/or Yoga researchers were purposively selected and were administered with a questionnaire with all the identified practices, electronically through email. The content validity was marked for each practice on a three-point scale (zero = not necessary, one = useful but not essential, two = essential), taking into account the safety aspect. CVR was calculated for each practice but only those with CVR ≥ 0.29 were included in the intervention.

CVR=(ne-N/2)/(N/2)

Where, ne = number of experts indicating “essential”

N = total number of experts.

Lawshe's content validity ratio (CVR) formula (33) was used to validate the content of the intervention. The final intervention consisted of daily 10-15 minutes of integrated yoga practices and additional 10 minutes of pranayama practice in the evening. Towards the validation of the drafted module, we included 25 Yoga experts (25 responders).  These highly qualified and experienced Yoga practitioners and/or Yoga researchers were purposively selected and were administered with a questionnaire with all the identified practices, electronically through email. The content validity was marked for each practice on a three point scale (zero = not necessary, one = useful but not essential, two = essential), taking  into account the safety aspect. CVR was calculated for each practice but only those with CVR ≥ 0.29 were included in the intervention.

CVR=(ne-N/2)/(N/2)

Where, ne = number of experts indicating “essential”

N = total number of experts.

**Table S1: Validation of yoga module for COVID-19 management**

| **Morning Practices** | **Ne** | **N** | **N/2** | **Ne-N/2** | **CVR** |  |
| --- | --- | --- | --- | --- | --- | --- |
| Preparatory practices |  |  |  |  |  |  |
| Spinal twisting (1 minute) | 11 | 20 | 10 | 1 | 0.1 | Not included |
| Forward and backward spinal flexion (1 min) | 12 | 20 | 10 | 2 | 0.2 | Not included |
| Hands in and out Breathing [5 rounds (2 min)] | 19 | 20 | 10 | 9 | 0.9 | Included |
| Hands Stretch Breathing [5 rounds (2 min)] | 19 | 20 | 10 | 9 | 0.9 | Included |
| Shoulder rotation (1min) | 19 | 20 | 10 |  | 0.9 | Included |
| Matsyasana/Sulabha Matsyasana (Fish pose) | 13 | 20 | 10 | 3 | 0.3 | Not included |
| Mukha dhouti (1 min) | 13 | 20 | 10 | 3 | 0.3 | Not included |
| Kapalabhati Kriya (30 strokes - 1 min) | 13 | 20 | 10 | 3 | 0.3 | Not included |
| Breathing practices/Pranayama |  |  |  |  |  |  |
| Abdominal breathing (3 min) | 17 | 20 | 10 | 7 | 0.7 | Included |
| Alternate nostril breathing (Nadishuddhi Pranayama (3 min) | 20 | 20 | 10 | 10 | 1 | Included |
| Bhramari Pranayama (2 min) | 20 | 20 | 10 | 10 | 1 | Included |
| Meditation (1 min)/ guided resolve | 18 | 20 | 10 | 8 | 0.8 | Included |
| **Evening practices** |  |  |  |  |  |  |
| Pranayama (above 3 practices): 10 min evening with guided resolve | 20 | 20 | 10 | 10 | 1 |  |

Overall CVR was found to be 0.70; practices with CVRs below 0.5 were not included in the main intervention (highlighted in yellow). Based on the above analysis, the final intervention module consisted of daily 15 minutes of  integrated yoga practices, with 5 min of 3 preparatory practices coordinated breathing with hands in and out , hand stretching and shoulder rotation) followed by diaphragmatic breathing (3 min), alternate nostril breathing (nadishuddhi Pranayama (3 min), and bhramari Pranayama (2 min). The sessions will be concluded with guided meditation with resolve (2 min).


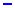


**APPENDIX I. ADDITIONAL RESULTS:**

**Table S2. Additional characteristics of the intention-to-treat population at baseline**

|  | **Overall (n=225)** | **Adjunct Tele yoga (n=113)** | **Standard of care (n=112)** |
| --- | --- | --- | --- |
| **Vital parameters** |  |  |  |
| Systolic blood pressure, mm Hg | 119.67±13.66 | 118.34±15.29 | 121.01±11.71 |
| Diastolic blood pressure, mm Hg | 78.70±7.797 | 78.38±7.53 | 79.02±8.077 |
| Heart rate, beats/min | 98.23±62.31 | 101.51±86.49 | 94.92±16.25 |
| Peripheral saturation O2, % | 95.20±3.67 | 95.28±3.49 | 95.12±3.85 |
| **Laboratory tests** |  |  |  |
| Creatinine, mg/dl | 0.78±0.71 | 0.77±0.93 | 0.79±0.36 |
| HbA1c, % | 6.30 () | 6.30 (2.45) | 6.30 (2.80) |

| Model a | Additionally adjusted for clinical status at day 1 and  symptom duration (continuous variable) | 2.08 (1.26-3.44) |
| --- | --- | --- |
| Model b | Additionally adjusted for baseline prevalence of breathlessness | 1.84 (1.09-3.12) |

**Table S3. Post-Hoc Sensitivity Analyses for the primary endpoint at 14^th^ day through ordinal regression**

Model a: Duration of symptoms from the onset to day 1 was included as a continuous covariate for the proportional odds model

Model b: Further adjusted for baseline prevalence of breathlessness, which was entered as a categorical variable in the model

**Table S4. Subgroup Analyses of the Primary Outcome**

| **Subgroup** | **Number of patients** | | **aOR (95% CI) for COVID Scale**  **score at 14 days** | **P-value for interaction** |
| --- | --- | --- | --- | --- |
|  | Adjunct Yoga  (n=113) | Standard of care  (n=112) |  |  |
| COVID outcome scale category at randomization |  |  |  |  |
| 3 | 54 (47.79) | 43 (38.39) |  | 0.955 |
| 4 | 57 (50.44) | 63 (56.25) | 2.04 (0.99-4.20) |  |
| 5 | 2 (1.77) | 6 (5.36) |  |  |
| Duration of symptoms before randomization |  |  |  |  |
| Highest tertile (5-9 days) | 39 (34.51) | 42 (37.50) | 2.60 (1.03-6.6)* | 0.569 |
| Middle tertile (3-4 days) | 33 (29.20) | 26 (23.21) | 2.63 (0.86-8.10) |  |
| Lowest tertile (0-2 days) | 41 (36.28) | 44 (39.28) | 1.19 (0.52-2.74) |  |
| Age, years |  |  |  |  |
| ≤43.5 | 66(58.41) | 57 (50.89) | 1.90 (0.94-3.83) | 0.497 |
| ≥43.5 | 47 (41.59) | 55 (49.11) | 2.49 (1.15-5.39)* |  |
| Gender |  |  |  |  |
| Male | 51 (45.13) | 51 (45.53) | 1.56 (0.77-3.19) | 0.175 |
| Female | 62 (54.87) | 61 (54.46) | 3.24 (1.47-7.13)* |  |
| Major comorbidities |  |  |  |  |
| Diabetes |  |  |  |  |
| Yes | 42 (37.17) | 43 (38.39) | 2.32 (0.98-5.53)* | 0.769 |
| No | 71 (62.83) | 69 (61.61) | 1.78 (0.93-3.40) |  |
| Hypertension |  |  |  |  |
| Yes | 21 (18.58) | 26 (23.21) | 1.35 (0.43-4.20) | 0.252 |
| No | 92 (81.42) | 86 (76.78) | 2.35 (1.30-4.22) |  |
| Coronary artery disease |  |  |  |  |
| CRP, mg/L |  |  |  |  |
| >26 | 55 (49.11) | 54 (48.65) | 1.81 (0.86-3.78) | 0.730 |
| ≤26 | 57 (50.89) | 57 (51.35) | 2.61(1.28-5.32) |  |
| LDH, U/L |  |  |  |  |
| ≤302 | 62 (54.86) | 51 | 1.54 (0.73-3.26) | 0.810 |
| >302 | 51 | 61 | 3.21(1.49-6.95) |  |

The adjusted odds ratios and 95% confidence intervals (CI). Adjusted only for age and gender. Adjustments for duration of symptoms before randomization and clinical scores at admission could not be considered as assumptions of proportional odds were violated. The cut off values (26 mg/L) for CRP is derived from the reports on prediction of progression to severe disease of COVID. ^1^

None of the interaction terms were found to be significant.

**Table S5: Clinical Status Outcomes (Intention-to-Treat Population).**

|  | **Total Subjects** | **Tele-yoga** | **Standard of care** | **Effect Estimate (95% CI) Proportional Odds Ratio** |
| --- | --- | --- | --- | --- |
| ***Secondary outcome: seven-level ordinal outcome at 7 days† (%)*** |  |  |  | 3.61 (2.13-6.10), P<0.001 |
| Distribution — no. (%) |  |  |  |  |
| 7: Not hospitalized with no limitations on activities | 49 (21.78) | 35 (30.97) | 14 (12.50) |  |
| 6: Not hospitalized but with limitations on activities | 112 (49.78) | 60 (53.10) | 52 (46.43) |  |
| 5: Hospitalized, not receiving supplemental oxygen | 48 (21.33) | 17 (15.04) | 31 (27.68) |  |
| 4: Hospitalized, receiving supplemental oxygen | 9 (4.00) | 0 (0) | 9 (8.03) |  |
| 3: Hospitalized, receiving noninvasive ventilation or high-Low nasal cannula | 2 (0.89) | 0 (0) | 2 (1.78) |  |
| 2: Hospitalized, receiving mechanical ventilation | - | - | - |  |
| 1: Death | 5 (2.22) | 1 (0.88) | 4 (3.57) |  |
| ***Secondary outcome: seven-level ordinal outcome at 28 days† (%)*** | **Total Subjects** | **Tele-yoga** | **Standard of care** | 1.70 (0.97-2.99), 0.07 |
| Distribution — no. (%) |  |  |  |  |
| 7: Not hospitalized with no limitations on activities | 156 (69.33) | 84 (74.34) | 72 (64.29) |  |
| 6: Not hospitalized but with limitations on activities | 48 (21.33) | 23 (20.35) | 25 (22.32) |  |
| 5: Hospitalized, not receiving supplemental oxygen | 12 (5.33) | 3 (2.65) | 9 (8.03) |  |
| 3: Hospitalized, receiving noninvasive ventilation or high-fow nasal cannula | 1 (0.44) | 0 (0) | 1 (0.89) |  |
| 1: Death | 8(3.57) | 3(2.65) | 5 (4.46) |  |

# Oxygen saturation measured by pulse oximetry (SpO_2_) ≥90% on room air at sea level. The moderate disease definition was based on respiratory rate  ≥ 15 < 30 and/or partial 90%–94%. Patients who had scores on other levels of the seven-level ordinal scale were not eligible. The difference in clinical status distribution on day 7 between the adjunct tele-yoga group and standard care groups was statistically significant

**Table S6. Biomarker levels at day 5 post randomization**

|  | **Median (Interquartile Range, IQR)** | | **P and F value** |
| --- | --- | --- | --- |
|  | **Adjunct Tele-Yoga (n=113)** | **Standard of care**  **(n=112)** |  |
| **Biomarkers of COVID 19** |  |  |  |
| C-reactive protein, mg/l | 8.11 (2.27-17.34) | 13.83 (5.23-27.91) | 0.001*12.35 |
| Lactate dehydrogenase U/L | 271(211.50-357.57) | 316.67 (253-412.60) | 0.029*, 4.90 |
| Ferritin, ng/ml | 233 (84.57-361) | 229.78 (138.85- 436) | 0.903, 0.015 |
| D-Dimer, ng/ml | 170 (90-335) | 231 (110-415.30) | 0.125, 2.37 |
| Il-6, ng/ml | 9.07 (2.72-32.37)  N’.26 (24.82-193.11) | 18. 18) | 0.994, 0.004 |
| **Other markers** |  |  |  |
| Ct Value | 33.44 (30.78-34.595) | 33.44 (32.16-34.71) | 0.77, 0.09 |
| Perceived stress scale score | 18 (14.42-22) | 18.58 (15-22) | 0.690, 0.160 |
| Duration of hospital stay -days | 10 (6.5-13) | 8 (6-12) | 0.300, 1.08 |
|  |  |  |  |
| **Kidney function** |  |  |  |
| Creatinine, mg/dl | 0.62 (0.50-0.74) | 0.62 (0.46-0.82) | 0.150, 0.209 |
| EGFR | 131.32 (109.68-135.38) | 120.13 (101.05-157.69) | 0.499, 0.460 |
| Hemoglobin, mg/dl | 13.75 (12.40-14.80) | 13.50 (12.30-14.60) | 0.911, 0.01 |
| Total Leukocyte count, 10^9^/L | 9.2 (6.4-12.27) | 8.9 (6.8-12.20) | 0.414, 0.671 |
| *Differential blood count* |  |  |  |
| Monocytes, % | 6.60 (4.65-9.20) | 7.95 (5.27- 10.60) | 0.154, 2.09 |
| Lymphocytes, % | 19.20 (10.05-30.35) | 19.40 (11.65-31.45) | 0.912, 0.01 |
| Neutrophils, % | 72.80 (58.22-83.08) | 70.15 (56.17-81.02) | 0.861, 0.03 |

eGFR, estimated glomerular filtration rate

**Figure S1.  Survival through 28 Days Following Randomization**

The survival curves are survival function (Kaplan-Meier) curves with a *P* value calculated by the log-rank test. Patients were followed up for death until 28 days following randomization using in-hospital records and telephone follow-up. There was no difference between the tele-yoga group and the standard of care group in survival (adjusted hazard ratio, 1.05 [95% CI, 0.60-1.85])

**Figure S2: Change in clinical status over time**


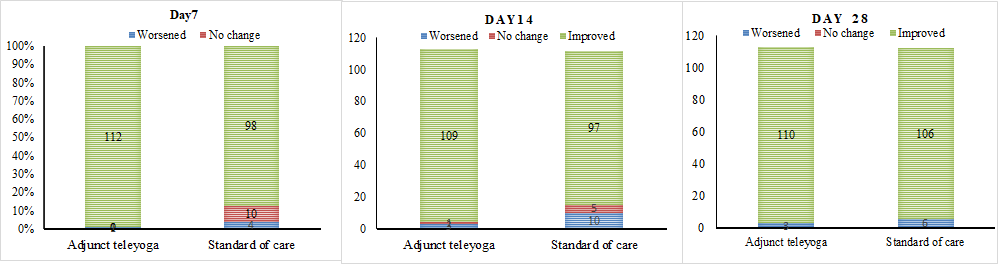


Change in outcomes at Day 7; P <0.001: Change Day 14; P value=0.245: Change Day 28; P value=0.338

The figure depicts the change in clinical status from baseline on days 7, 14, and 28 for the adjunct yoga treatment and the standard of care groups according to the 7-point ordinal scale. Patients who worsened had a loss of at least 1 point and patients who improved had an increase of at least one point. The ordinal scale categories are as follows: 1, death; 2, receiving invasive mechanical ventilation; 3, receiving high-flow oxygen; 4, receiving low-flow oxygen; 5 or 6, breathing ambient air; and 7, discharge

**Table S7. Adverse events**

|  | Adjunct tele yoga  (n=113) | Standard of care  (n=112) |
| --- | --- | --- |
| Events |  |  |
| Death, n (%) | 3 (2.65) | 5 (4.46) |
| Extension of hospitalization — no (%) | 12 (10.62) | 21 (18.75) |
| Sinus tachycardia (resolved), no. (%) | 1 (0.88) | 0 (0) |
| Pulmonary embolism — no. (%) | 1 (0.88) | 0 (0.89) |
| Pneumothorax — no. (%) | 1 (0.88) | 1 (0.89) |
| Elevated ALT or AST level — no. (%)^§^ | 3 (2.65) | 3 (2.67) |
| Acute renal injury, n (%) | 0 (0) | 1 (0) |

Additional references:

Valeri L, Vanderweele TJ. Mediation analysis allowing for exposure-mediator interactions and causal interpretation: theoretical assumptions and implementation with SAS and SPSS macros [published correction appears in Psychol Methods. 2013 Dec;18(4):474]. *Psychol Methods*. 2013;18(2):137-150. doi:10.1037/a0031034

Schmidt WP. Randomised and non-randomised studies to estimate the effect of community-level public health interventions: definitions and methodological considerations. Emerg Themes Epidemiol. 2017;14:9. Published 2017 Sep 7. doi:10.1186/s12982-017-0063-5

Wang G, Wu C, Zhang Q, et al. C-Reactive Protein Level May Predict the Risk of COVID-19 Aggravation. Open Forum Infect Dis. 2020;7(5):ofaa153. Published 2020 Apr 29. doi:10.1093/ofid/ofaa153
